# Phenotypic approach artemisinin resistance in malaria rodent as *in vivo* model

**DOI:** 10.14202/vetworld.2017.790-797

**Published:** 2017-07-19

**Authors:** Lilik Maslachah, Thomas V. Widiyatno, Lita Rakhma Yustinasari, Hani Plumeriastuti

**Affiliations:** 1 Department of Basic Medicine, Veterinary Pharmacy Laboratory, Faculty of Veterinary Medicine, Airlangga University Surabaya, Indonesia; 2 Department of Pathology, Faculty of Veterinary Medicine, Airlangga University Surabaya, Indonesia; 3 Department of Veterinary Anatomy, Faculty of Veterinary Medicine, Airlangga University Surabaya, Indonesia; 4 Department of Veterinary Pathology, Faculty of Veterinary Medicine, Airlangga University Surabaya, Indonesia

**Keywords:** artemisinin, parasite clearance time, phenotypic, *Plasmodium berghei*, recrudescence time, resistance

## Abstract

**Aim:**

The aim of this study is to prove the development of artemisinin resistance phenotypically in malaria rodent as an *in vivo* resistance development model in humans.

**Materials and Methods:**

*Plasmodium berghei* was infected intraperitoneally in mice, then artemisinin was given with “4-day-test” with effective dose (ED) 99% dose for 3 days which begins 48 h after infection (D2, D3, and D4). Parasite development was followed during 5^th^ until 10^th^ days of infection. After parasitemia >2% of red blood cell which contains parasites on 1 mice, that mice were used as donor to be passaged on the new 5 mice. After that, parasitemia was calculated. ED_50_ and ED_90_ were examined with parasite clearance time (PCT), recrudescence time (RT), and also morphology development examination of intraerythrocytic cycle of *P. berghei* with transmission electron microscope.

**Results:**

Among the control group compare with the treatment group showed significant differences at α=0.05 on 5^th^ day (D5) until 10^th^ day (D10). The control group of 4^th^ passage (K4) with passage treatment group of 4^th^ passage (P4) on the 10^th^ days (D10) post infection showed no significant differences in the α=0.05. The average percentage of inhibition growth was decreasing which is started from 5^th^ to 10^th^ day post infection in P1, P2, P3, and P4. On the development of *P. berghei* stage, which is given repeated artemisinin and repeated passage, there was a formation of dormant and also vacuoles in *Plasmodium* that exposed to the drug.

**Conclusion:**

Exposure to artemisinin with repeated passages in mice increased the value of ED_50_ and ED_90_, decreased the PCT and RT and also changes in morphology dormant and vacuole formation.

## Introduction

Malaria is still a public health problem in more than 90 countries. A rapid increasing incidence of morbidity and mortality of malaria is caused by increasing parasite resistance to antimalarial drugs. A new drug for malaria treatment which is used until right now is artemisinin and its derivatives; this drug has the effect of working faster than other antimalarial drugs because they have more complex mechanisms of action. However, there have been indicated that the *Plasmodium* parasite has been resistant to this drug [1]. Clinical results already shown in two patients infected with *Plasmodium falciparum* that was resistant to artesunate in Cambodia [2]. Results of the research show a decrease in efficacy against malaria falciparum to the combination of artesunate-mefloquine in Cambodia [3].

Results of *in vitro* studies on *P. falciparum* which is exposed with repeated artemisinin as antimalarial drug showed an increase of 50% inhibitory concentration (IC_50_), phenotypic changes dormant, and faster growth after *Plasmodium* viable from a dormant form. Besides, the exposure to artemisinin also causes mutations in genes *pfatpase6* [4]. The presence of parasite pressure on the use of drugs with subcurative doses will lead to the development of new parasite that can survive on the drug. The results of this research become an emergency because it could be developed resistance in human being and lead to be one of health problems in the world because there is no substitute for a new drug artemisinin. Malaria treatment failure using antimalarial drug artemisinin and its derivatives appears to be an era of untreatable malaria.

*In vivo* experimental studies using rodent malaria used to support the translation of laboratory studies into clinical studies, because the spectrum of malaria in humans is not yet clearly understood how the mechanism of the pathogenesis. So that, this study could be used to explain the mechanisms of resistance to artemisinin *in vivo* using mice as an animal model that infected with *Plasmodium berghei*. Resistance of malaria and developed resistance to antimalarial drugs need to do research to develop effective control strategies for malaria. However, this research is really difficult to conduct in endemic areas because of the many confounding factors such as infection multiple clones of infective mosquito bites that spreading. This research also impossible to do in humans because of ethical reason [5]. This study used rodent malaria as a model of resistance *in vivo* in humans by doing exposure to *P. berghei* with artemisinin on effective dose 99% (ED_99_: 200 mg/kg weight of mice) through repeated passage in mice. Exposure of artemisinin as antimalarial drug with repeated passage *in vivo* in mice can be used as a basic to predict and anticipate the spread of artemisinin antimalarial drug resistance in practical use in the clinic.

## Materials and Methods

### Ethical approval

This study was conducted after getting approval with certificate number no. 464 KE from the Animal Ethics Committees of Faculty of Veterinary Medicine, Airlangga University Surabaya Indonesia.

### Parasites, host, and drugs that used in the study

A parasite which is used to infect mice is *P. berghei* ANKA strain. Mice which are used are male Albino Swiss strain, the weight is 20-30 g, and the aged is 2.5 months. Artemisinin which is used pro analysis from Sigma Chemical Co.

### Infection dose of P. berghei in mice

Mice is infected with red blood cells (RBCs) containing parasites 1×10^5^ *P. berghei* in 0.2 ml intraperitoneally. To determine the infection has occurred in mice, microscopic examination of erythrocytes of mice was done every day with thin blood smears that taken from tail vein of mice and stained with Giemsa 20% [6].

### Selection of artemisinin antimalarial drug resistance in vivo in mice

Exposure to artemisinin antimalarial drug in the treatment group: After inoculation of RBCs containing parasites 1×10^5^ *P. berghei* in 0.2 ml on 5 mices (D0) and then given artemisinin antimalarial drug with “4-day-test” with ED_99_ dose (200 mg/kg weight of mice) was given for 3 days started at 48 h after infection (D2). Parasitemia was monitored and calculated at 120 h after infection. After parasitemia >2% of RBCs containing parasites, they are used as donor and was passaged on new 6 mice. After 48 h post infection, the mice were exposed to artemisinin antimalarial drug with the same ED_99_ dose for 3 consecutive days 4 times passages. Control group: After inoculation of RBCs containing parasites 1×10^5^ *P. berghei* in 0.2 ml at 6 mice (D0) was given no medication, parasitemia monitored and calculated at 48 h after infection. After parasitemia >2% of RBCs containing parasites, they are used as donor and was passaged on the new 5 mice, and the passages were repeated on mice 4 times. The development of parasite was followed until 10^th^ day of infection in all treatments [7,8].

### Parasitemia calculation

Calculation of parasitemia in mice for each exposure to artemisinin and every passage in mice conducted after 120 h (D5) post infection. Thin smear of blood vessels from tail vein of mice is made, then fixed with methanol, stained with Giemsa 20% for 20 min, then washed with water and dried. After that, the percentage of parasitemia of *P. berghei* was calculated by counting the number of infected erythrocytes per 1000 erythrocytes under a light microscope with 1000x magnification [9,10].

### Measurement of 50% and 90% ED level (ED_50_ and ED_90_)

Measurement of ED_50_ and ED_90_ level for each exposure to the artemisinin antimalarial drug in mice was counted every passage 120 h (D5) post infection using the formula: (A−B)/A)×100

Where, A is the average parasitemia in control group and B is parasitemia in treatment group. Determination ED_50_ and ED_90_ is calculated using a linear regression program [11].

### Examination of parasite clearance time (PCT) and recrudescence time (RT) of P. berghei

Examination of PCT and RT *P. berghei* was done by checking the growth of the parasite 48 h after completion of treatment for 3 days or 120 h (D5) post infection which is showed by the absence of parasites in the thin blood smear of mice that taken from a tail vein and stained with Giemsa 20% for 20 min and examined using a light microscope with 1000× magnification and followed every day to see the development until 10^th^ day post infection until discovered a parasite >5% that can grow back (RT) [12].

### Morphological stadium observation of P. berghei development

Morphological stage observation of the intraerythrocytic cycle development of *P. berghei* ring, trophozoites and schizonts in the control group and the treatment of exposure to artemisinin-dose ED_99_ with repeated passages in mice was conducted every 48 h on 5^th^, 6^th^, 8^th^, and 10^th^ day post infection by counting the number of development dormant, ring, trophozoites and schizonts stage in thin blood smears that stained with 20% Giemsa for 20 min and examined using light microscope with 1000× magnification [13,14].

### Ultrastructural morphology observation with a transmission electron microscope (TEM)

RBC washed with sodium cacodylate pH 7.4, 500 mL and fixed with 5% glutaraldehyde containing cacodylate buffer pH 7.4 and 3% sucrose for 24 h (stored at a temperature of 4°C). Rinsed with sodium cacodylate 0.1 M pH 7.4 for 15 min and fixation is using osmium tetraoxide 2% and potassium ferricyanide K_3_Fe(CN)_6_ in 0.1 M cacodylate buffer, then dehydrated with gradual concentration of ethanol. Then, tissue is immersed back with a solution of pure Spurr and entered in a vacuum incubator 70°C overnight. This preparation will result tissue block with hard consistency. Tissue is cut with diamond knife with 40-55 nm thick and attached to the grid which has been coated with formvar 5% in chloroform and consists of 200 mesh. Results of pieces were stained with uranyl acetate, followed with triple lead then examined using a JEOL 1010 TEM. Morphology of *P. berghei* parasites in erythrocytes that have been exposed to artemisinin was observed and compared with negative control of *P. berghei* (without drug exposure) [15].

### Statistical analysis

The data on parasitemia percentage and growth inhibition of *P. berghei* were processed with two-way ANOVA with the level of significance set at 5% to determine differences in treatment. The data ED_50_ and ED_90_ level, PCT, and RT of *P. berghei* were analyed with linier regression using SPSS 17.0 and morphology *P. berghei* developmental stage was analyzed with description

## Results

### Results of parasitemia percentage and growth inhibition of P. berghei in the repeated passage on the D5-D10 post infection after being given artemisinin for 3 days in the 2^nd^ day post infection

**Figure-1:**
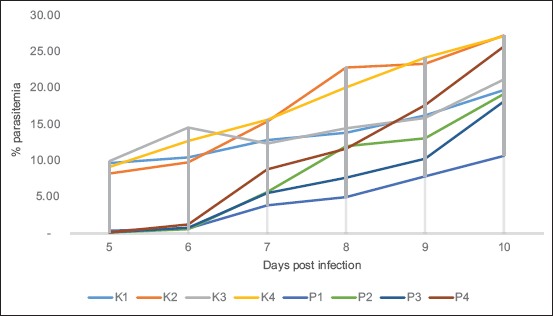
Graphic of *Plasmodium berghei* parasitemia percentage which is repeated passage on D5- D10 after treated artemisinin for 3 days in D2 post infection. K1: Control once passage untreated, K2: Control twice passage untreated, K3: Control 3 times passage untreated, K4: Control 4 times passage untreated P1: Once treated and once passage, P2: Twice treated and twice passage, P3: 3 times treated and 3 times passage, P4: 4 times treated and 4 times passage.

Percentage of parasitemia of *P. berghei*, which is repeated passage in D5-D10 after being given artemisinin for 3 days in D2 post infection showed that among the control group (K1 to K4) and the treatment group (P1 to P4) on the repeated passage (1^st^ passage to 4^th^ passage) showed significant differences in the α=0.05 on day 5 (D5) up to day 10 (D10) post infection except in the 4^th^ passage control group (K4) with 4^th^ passage treatment group (P4) on 10^th^ day (D10) post infection showed no significant differences in the α=0.05. That results are tested with the average difference test and two tail t-test. The results of this study also showed that *P. berghei* infection with repeated passage (P1, P2, P3, and P4) in mice that were given artemisinin repeatedly showed a decrease of % growth inhibition (Figure-1).

### Measurements ED_50_ and ED_90_ level P. berghei that repeated passages on the D5-D10 after being given artemisinin for 3 days in D2 post infection

**Figure-2:**
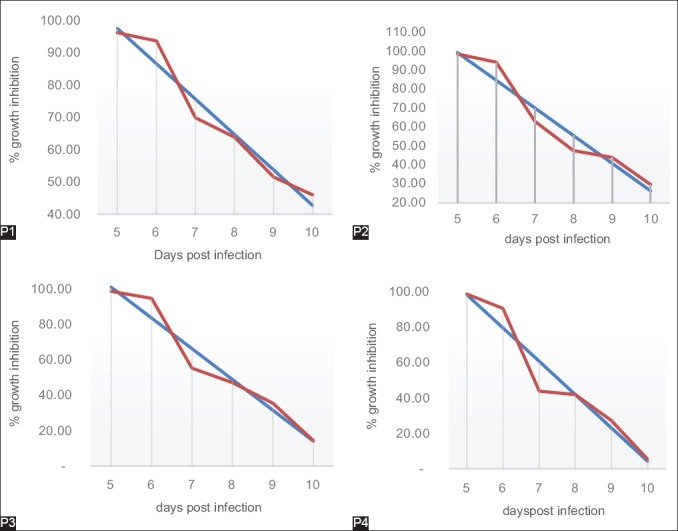
Graphic of linear regression of 50% and 90% effective dose level *Plasmodium berghei* that repeated passages on the D5-D10 after treated artemisinin for 3 days in D2 post infection. P1: Once treated and once passage, P2: Twice treated and twice passage, P3: 3 times treated and 3 times passage, P4: 4 times treated and 4 times passage.

Linear regression test is known that ED_50_ and ED_90_ *P. berghei* in P1 ED_50_ on 9.3^th^ days and ED_90_ on 5.7^th^ days with the regression equation. Y=152.41−10.96 X. On P2 ED_50_ on 8.3^th^ days and ED_90_ on 5.6^th^ with the regression equation Y=172.41−14.62 X. On P3 ED_50_ on 7.9^th^ days and ED_90_ on 5.6^th^ days with the regression equation Y=187.78−17.37 X. On P4 ED_50_ on 7.5^th^ days and ED_90_ on 5.4^th^ days with the regression equation Y=192.13−18.8 X (Figure-2).

### PCT and RT P. berghei that repeated passages on the D5-D10 after being given artemisinin for 3 days in D2 post infection

**Figure-3:**
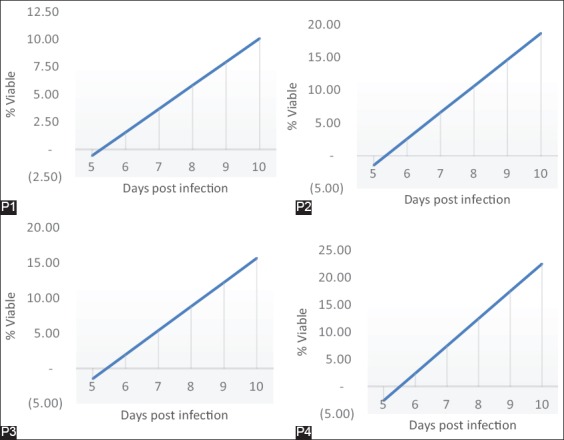
Parasite clearance time and recrudescence time *Plasmodium berghei* that repeated passages on the D5-D10 after treated artemisinin for 3 days in D2 post infection. P1: Once treated and once passage, P2: Twice treated and twice passage, P3: 3 times treated and 3 times passage, P4: 4 times treated and 4 times passage.

Artemisinin that given for 3 days in D2 post infection, then after reaching parasitemia 2% was passage to the new mice and given repeated artemisinin with the same dose up to 4 times passage shows PCT after 3 days of artemisinin treatment with dose 200 mg/kg body weight of mice on D5% parasitemia in P1 is approximately 0.362, P2 0.120, P3 0.140, and P4 0.140. RT *P. berghei* is counted after parasitemia reach 5% after treatment for 3 days. The results of RT on P1 parasitemia reach 5% after 7.7 days with the equation of regression is Y=−11.22+2.13 X. P2 parasitemia reach 5% after 6.61 days with the equation of regression is Y=−21.55+4.02 X. P3 parasitemia reach 5% after 6.9 days with the equation of regression is Y=−18.63+3.43 X. P4 parasitemia reach 5% after 6.5 days with the equation of regression is Y=−27.56+5.03 X (Figure-3).

### Morphology P. berghei that passage repeatedly after having been given artemisinin for 3 days in D2 post infection

**Figure-4:**
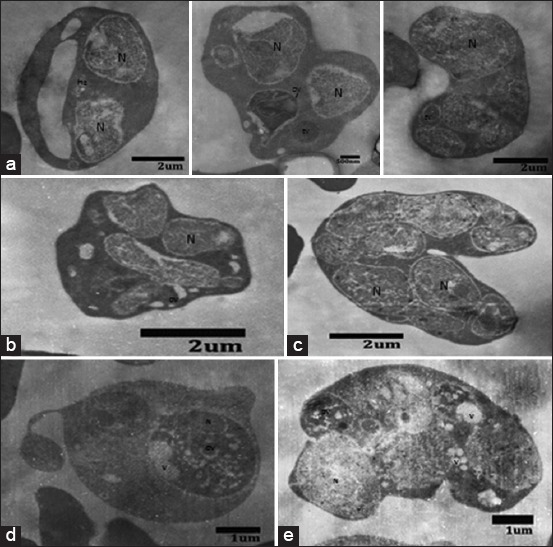
Morphology of *Plasmodium bergei* with transmission electron microscope on control and treatment artemisinin groups. N: Nucleus, V: Vacuole, DV: Digestive vacuole. (a) Control untreated, (b) once treated and once passage, (c) twice treated and twice passage, (d) 3 times treated and three times passage, (e) 4 times treated and 4 times passage.

Morphology of *P. berghei* with TEM control and treatment groups (Figure-4).

### Morphology of P. berghei developmental stages that passage repeatedly on D5-D10 after having been given artemisinin for 3 days in D2 post infection

**Figure-5:**
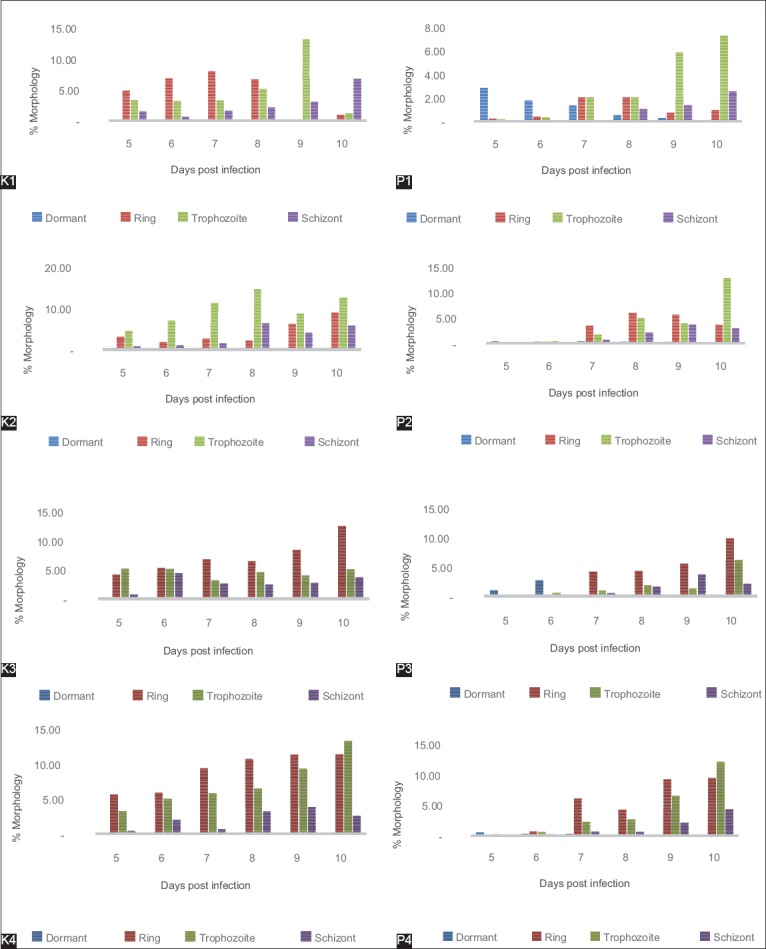
Morphology of *Plasmodium berghei* developmental stages which repeated passage on D5-D10 after treated artemisinin for 3 days in D2 post infection. K1: Control once passage untreated, K2: Control twice passage untreated, K3: Control 3 times passage untreated, K4: Control 4 times passage untreated P1: Once treated and once passage, P2: Twice treated and twice passage, P3: 3 times treated and 3 times passage, P4: 4 times treated and 4 times passage.

The description of developmental stages of *P. berghei* which passage repeatedly on D5-D10 after having been given artemisinin for 3 days in D2 post infection showed that in the control group which only infected with *P. berghei* did not show any formation dormant in all of the control group that passaged repeatedly while in the treatment group that infected with *P. berghei* and treated artemisinin for 3 days in D2 post infection, there was a formation of dormant (Figure-5).

## Discussion

### Results of parasitemia percentage and inhibition growth of P. berghei that passaged repeatedly on D5-D10 post infection after being given artemisinin for 3 days in D2 post infection

The percentage of parasitemia in *P. berghei* that passages repeatedly on the D5-D10 after having been given artemisinin for 3 days in D2 post infection show decreasing percentage of parasitemia when compared with the control group. Artemisinin can decrease the parasite significantly within 24-48 h after treatment and more potent than other antimalarials drugs, but artemisinin and its derivatives have t_½_ elimination in 1 h so that is unable to eliminate the parasite after 3 days of treatment. Therefore, artemisinin should be combined with other drugs such as amodiaquin, piperaquin, etc., to extend the working time of the medicine (duration of action) so that the recrudescence after administration of artemisinin can be avoided [16].

Repeated passage of *P. berghei* up to 4 times after have been given artemisinin showed an increased percentage of parasitemia in the treatment group which is showed by significant differences between the treatment groups that passage 1 times, 2 times, 3 times, and 4 times. This suggests that the parasite is viable after drug exposure more than once showed development toward resistant by the image of an extension of PCT and increased of speed recrudescence [17]. This is shown by the results % inhibition growth that decreases continually and increases the growth rate in the treatment group that passaged repeatedly.

The results of this research on the 4^th^ passage of control group 4 (K4) with 4^th^ passage of treatment group (P4) on 10^th^ day (D10) post infection showed no significant difference with the control group which were not given artemisinin at α=0.05. This suggests that the growth rate of the treatment group which was given repeated artemisinin up to 4 times with the same dose for each passage is not able to inhibit parasite growth with the same dose. The results of *in vivo* studies using mice as a model to be infected with *P. berghei* is consistent with *in vitro* research that is using *P. falciparum*, and the result showed an increasing value of IC_50_ for each repeated exposure to artemisinin which means that inhibit 50% of parasite requires a higher dose than the dose of artemisinin earlier [18].

### Results of measurements ED_50_ and ED_90_ level P. berghei that passages repeatedly on the D5-D10 after being given artemisinin for 3 days in D2 post infection

Results of linear regression test are known that ED level ED_50_ and ED_90_ *P. berghei* after repeated exposure of artemisinin in the repeated passage and given artemisin on the same dose for each passage showed an increasing of ED_50_ and ED_90_ which is to inhibit parasite growth in the same time. The results indicate that the ED of artemisinin to inhibit *P. berghei* growth is increasing by shortening of the required time for the parasite to grow back so that the parasites require higher doses to be able to inhibit its growth in the same time.

The results are consistent with research with the selection of resistant *P. berghei* to pyronaridine by repeated passage 20 times for 6 months. The results showed ED_50_ and ED_90_ increased from 40 to 66 time [11]. The results are consistent with research in *P. falciparum* F32 Tanzania strain that exposed to artemisinin for 3 years with low concentrations 0.01 µM, and then, concentrations are increased up to 10 µM for 100 exposure times. The results after selection of F32-ART strain showed that F32-ART with higher artemisinin exposure (35 and 70 µM) for 96 h, only on F32-ART strain that has been selected will able to survive [19]. Other studies from the results of research in *P. falciparum* GC06 and CH3-61 strains before and after selection with artemisinin with increased concentrations of each of 0-20 and 0-100 nM, after the parasite is viable, its is showed an increasing IC_50_ values on the strains after selection with artemisinin which is the first GC06 strain has IC_50_ value from 3.1±0.1 changed to 12.5±1.6 nM and the first CH3-61 strains have IC_50_ values from 28.8±1.3 changed to 58.3±4.5 nM [16].

Research conducted by Tucker *et al*. [20] also showed that the parasite that has been resistant required greater concentrations of the drug to inhibit parasite growth compared to its stem. IC_50_ has increased in the resistant parasite compared with parasitic stem on artemisinin, which is described as follows: Stem of W2 strain has a value of IC_50_ 1.3±071 ng/ml, resistant W2QSH200x2 strain have IC_50_ values 4.2±2.2 ng/ml, stem of D6 strain has IC_50_ value 0.92±0.10 ng/ml, resistant D6QSH2400x5 strain have IC_50_ value 8.8±1.0 ng/ml and the stem of TM91c235 strain showed IC_50_ values 2.2±1.8 ng/ml, and resistant TM91c235AL280x2 strain have IC_50_ value 8.7±5.4 ng/ml. This means that resistant parasites have an ability to withstand in higher drug induction.

Increasing the value of IC_50_ become 2-5 times also apply during three parasite strains that have been tolerant to acid artelinic, changes in the value of IC_50_ were also followed with an increasing in the number of copies, the expression of mRNA, and protein expression of *pfmdr1* genes [21].

### Examination of PCT and RT P. berghei that passaged repeatedly on the D5-D10 after being given artemisinin for 3 days in D2 post infection

The provision of artemisinin for 3 days in D2 post infection, then after reaching parasitemia 2% was passages to the new mice and given artemisinin repeatedly with the same dose 4 times passage shows PCT after 3 days of artemisinin treatment dose of 200 mg/kg body weight of mice on D5 showed an extension time of PCT and accelerate RT. It was shown from the results that the PCT in P1 ranging from 0.362, P2 0.120, P3 0.140, and P4 0.140 with dormant morphology. RT *P. berghei* is calculated after parasitemia reach 5% after receiving treatment for 3 days. The results of RT on P1 after 7.7 days, P2 after 6.61 days, P3 after 6.9 days, and P4 after 6.5 days; the results are consistent with research conducted by Teuscher *et al*. [22] that treatment with dormant form of artesunate from ring stadium is expected 0.001-1313 to grow back. Recovery from dormant parasite is a time to reach 5% parasitemia in the form of dormant. This is also found in the mice. From the results of research conducted by La Crue *et al*. [12] shows that the form of dormant ring began recrudescence about 7-9 days. RT is consistent with the results of research which the ranges are 7.7 days post infection and the time that required is shorter after 2^nd^, 3^rd^ and 4^th^ times of passage.

The overview morphology of dormant in *P. falciparum* which exposed to artemisinin antimalarial drug is a defence mechanism for the parasite to be able to survive from the exposure to artemisinin antimalarial drug. Parasites will be able to grow normally after the drug pressure is removed. In this dormant period, the parasite can survive in a few days by slowing down the process of metabolism to limit the effects of the drugs because there is no DNA synthesis in this situation [19].

This results are consistent with research conducted by Tucker *et al*. [20] on *P. falciparum* D6 stem strain with *P. falciparum* in strain that has been resistant D6QSH2400x5 showed normal morphology after exposure to artemisinin antimalaria, require faster time to grow back to normal and the ratio of the morphology of normal parasites two times higher in the parasite which has been resistant when compared with the stem parasitic strains. This shows that the strain of parasite that has been already resistant to artemisinin have an ability to produce more dormant parasites and can be faster to get out from dormant period (viable) so that the parasites are already resistant to artemisinin have the speed of recovery is higher than the stem strain which are not resistant so it will accelerate its recrudescences.

### Result of observations of morphology P. berghei that passage repeatedly after having been given artemisinin for 3 days in D 2 post infection

The description of developmental stages of *P. berghei* which passage repeatedly on D5-D10 after having been given artemisinin for 3 days in D2 post infection showed that in the control group, which only infected with *P. berghei* did not show any formation dormant in all of the control group that passaged repeatedly while in the treatment group that infected with *P. berghei* and given artemisinin for 3 days in D2 post infection, there were formations of dormant. The ability of the parasite in this dormant period as a resistance mechanism that leads to recrudescences of parasites and extension of PCTs.

The mechanism of artemisinin induces the formation of dormant is still unclear. However, it is believed that the existence of dormant stage is associated with cell cycle regulation such as cyclin-dependent kinase and cyclins. This dormant overview is also reported by Teuscher *et al*. [23] and Witkowski *et al*. [19]. Decreasing in metabolic activity on the stage of the ring as a prerequisite of the ability of resistant parasite to be a form of dormant on the artemisinin drug administration, so that the phenomenon can be used to explain the resistance to artemisinin is an increasing of parasites in the form of dormant (quiescence) from the ring in exposure to artemisinin antimalaria drug. Therefore, killing the resistant parasite required greater concentration of artemisinin antimalarial drug. If the concentration of the drug is same, the parasite is still able to survive and breed back with a faster time.

Ultrastructure by TEM on the ring stage that treated for 24 h with artemisinin showed a loss of substance of the membrane so that the crystal hemozoin is located in the cytoplasm of the parasite, and there was a formation of vacuoles. The trophozoites stage which was treated with a high concentration of artemisinin for 4 to 8 h, showed loss of digestive vacuoles integrity, has an ability to alkylate the protein and lipid components of digestive vacuole membrane. In the schizonts stage, there was merozoites morphology with abnormal nuclei. This condition has led to decrease *Plasmodium* parasitemia due to death or inhibition in the development stage by exposure to artemisinin antimalarial drug [16].

## Conclusion

The results of this study can be concluded that artemisinin exposure with repeated passages in mice caused an increasing of ED_50_ and ED_90_ values. Decreasing PCT and RT and morphological changes in intraerythrocytic cycle, there was a dormant formation and loss of substance from the digestive vacuole membrane so that the crystal hemozoin is located in the cytoplasm of the parasite and there was a formation of vacuoles.

## Authors’ Contributions

LM: Research project leader and coordinating research, designed study, analyzed data, drafted paper and corresponding author. TVW: Examination of PCT and RT. LRY: Processing of blood for morphological stadium observation and HP: Processing of blood for TEM. All authors read and approved the final manuscript.
